# Temporal Grading Index of Functional Network Topology Predicts Pain Perception of Patients With Chronic Back Pain

**DOI:** 10.3389/fneur.2022.899254

**Published:** 2022-06-10

**Authors:** Zhonghua Li, Leilei Zhao, Jing Ji, Ben Ma, Zhiyong Zhao, Miao Wu, Weihao Zheng, Zhe Zhang

**Affiliations:** ^1^Department of Rehabilitation Medicine, Gansu Provincial Hospital of TCM, Lanzhou, China; ^2^Gansu Provincial Key Laboratory of Wearable Computing, School of Information Science and Engineering, Lanzhou University, Lanzhou, China; ^3^Department of Rehabilitation Medicine, The Second Affiliated Hospital of Xi'an Jiaotong University, Xi'an, China; ^4^Key Laboratory for Biomedical Engineering of Ministry of Education, College of Biomedical Engineering and Instrument Science, Zhejiang University, Hangzhou, China; ^5^Institute of Brain Science, Hangzhou Normal University, Hangzhou, China; ^6^School of Physics, Hangzhou Normal University, Hangzhou, China

**Keywords:** chronic back pain (CBP), dynamic functional connectivity, temporal grading index (TGI), pain assessment, depression

## Abstract

Chronic back pain (CBP) is a maladaptive health problem affecting the brain function and behavior of the patient. Accumulating evidence has shown that CBP may alter the organization of functional brain networks; however, whether the severity of CBP is associated with changes in dynamics of functional network topology remains unclear. Here, we generated dynamic functional networks based on resting-state functional magnetic resonance imaging (rs-fMRI) of 34 patients with CBP and 34 age-matched healthy controls (HC) in the OpenPain database *via* a sliding window approach, and extracted nodal degree, clustering coefficient (CC), and participation coefficient (PC) of all windows as features to characterize changes of network topology at temporal scale. A novel feature, named temporal grading index (TGI), was proposed to quantify the temporal deviation of each network property of a patient with CBP to the normal oscillation of the HCs. The TGI of the three features achieved outstanding performance in predicting pain intensity on three commonly used regression models (i.e., SVR, Lasso, and elastic net) through a 5-fold cross-validation strategy, with the minimum mean square error of 0.25 ± 0.05; and the TGI was not related to depression symptoms of the patients. Furthermore, compared to the HCs, brain regions that contributed most to prediction showed significantly higher CC and lower PC across time windows in the CBP cohort. These results highlighted spatiotemporal changes in functional network topology in patients with CBP, which might serve as a valuable biomarker for assessing the sensation of pain in the brain and may facilitate the development of CBP management/therapy approaches.

## Introduction

Pain and pain-related diseases are major contributors to disability (1–3). Among them, chronic back pain (CBP) is particularly prevalent with the disability rate increasing by over 54% in the last 30 years (4). CBP is known to be aroused by peripheral and central sensitization (5, 6), and alters the connectomics of the brain. Advances in neuroimaging technology have allowed researchers to characterize structural and functional alterations in the brain of patients with CBP (7–9), investigate brain signatures for predicting pain intensity (e.g., patterns of brain activation) (10–12), and identify effective methods for pain relief (13). Although these studies have painted a relatively comprehensive picture from abnormal brain alterations of CBP to its intervention, the relationship between spatiotemporal dysfunction of brain topological organization and pain intensity, a key question to the understanding of CBP, remains unclear.

The cerebral alterations of patients with CBP have been investigated in a series of studies focusing on functional connectivity (FC) established *via* functional magnetic resonance imaging (fMRI) (14–16). The large-scale functional network established by measuring pair-wise FC provides a comprehensive description of interactions among distinct brain regions, in terms of correlation, coherence, and topological organization (17–20). For example, chronic pain was associated with abnormal changes in the connectivity within the salience and central executive networks (21, 22) and altered strength of hub regions (23); and the study shows that pain would cause an increase in the shortest path length and clustering coefficient, and decreases in small-worldness (24). These alterations in inter-regional connectivity and network topology have largely affected the ability of information integration and segregation in the brain of patients with chronic pain (25, 26).

Existing evidence has suggested that the perception of pain was influenced by maladaptive neuroplastic changes over time (27, 28), and the CBP may derive from these changes in the central nervous system which could enhance nociceptive efficiency, influence normal attentional processing, and create the maladaptive perception of pain (29, 30). Since the “dynamic pain connectome” theory posits that the processing of pain in the brain is a dynamic process (31), it is necessary to investigate the CBP-related brain functional alteration from a time-varying perspective. Compared to traditional FC analysis, dynamic functional connectivity (dFC) is able to capture the alterations of intrinsic FC over time under various physiological and pathological brain conditions (32–36). For example, dynamic reconfiguration of functional brain networks was found during executive cognition by using dFC technology (37). Thus, dFC can provide additional information that may promote our understanding of the association between altered brain functions and CBP (38, 39). Recent studies have shown that dFC can reflect pain conditions at multiple timescales (e.g., short-term state and long-term trait) rather than just the current state of patients with chronic pain (40), and characterize pain pathophysiology from a dynamic perspective representing oscillations of the FC (41–43). However, previous studies have mainly focused on pain-related alterations in dFC, with few studies exploring how dynamics of functional network topology change in patients with CBP, and whether these changes can predict pain intensity has not been well explored.

The present study aims to investigate whether CBP is associated with dynamic changes in the functional network topology and to find an effective feature that could accurately predict the intensity of pain in the brain of patients with CBP. Resting-state fMRI data of 34 patients with CBP and 34 age-matched healthy controls (HC) were used to estimate the dFC through a sliding window approach along the time sequence. Degree centrality, clustering coefficient (CC), and participation coefficient (PC) of dFC network were calculated at each time window and cascaded to represent the dynamic fluctuation of network topology from the perspectives of nodal importance, local efficiency, and modular communication, respectively. Temporal grading index (TGI), a new feature that quantifies the oscillation slope of each network metric of the CBP cohort relative to the normative oscillation sequence of the HCs, was proposed and utilized to predict the pain intensity of the patients. TGI of these network metrics were submitted to three commonly used regression models (i.e., support vector regression [SVR], least absolute shrinkage and selection operator [Lasso], and elastic net), with a 5-fold cross-validation strategy, to examine the effectiveness of dynamic network topology on explaining pain intensity of patients with CBP.

## Materials and Methodology

### Participants

In the study, MRI data of 34 patients with CBP and 34 healthy controls (HC) who had matched age and gender in patients with CBP were downloaded from the Open Pain database (www.openpain.org). The database was collected by the OpenPain Project (OPP) for scientific investigation, teaching, or the planning of clinical research studies. All patients with CBP have completed the Short-Form of the McGill Pain Questionnaire (SF-MPQ), including a visual analog scale (VAS) ranging from 0 (painless) to 10 (maximum imaginable pain). The Beck Depression Inventory (BDI) was used to access the depression scores of all participants. Questions of SF-MPQ and BDI were finished 1 h before the brain scan.

### MRI Data Acquisition

MRI data were acquired on a 3-Tesla Siemens Trio whole-body scanner using the standard radio-frequency head coil. All participants were required to close their eyes during the scan. High-resolution 3-dimensional T1-weighted data were acquired with the following parameters: voxel size 1 × 1 × 1 mm^3^, repetition time (TR) = 2,500 ms, echo time (TE) = 3.36 ms, flip angle = 9°, number of slices = 160, field of view = 256 mm, in-plane matrix resolution = 256×256. Resting-state fMRI (rs-fMRI) data were acquired using an echo-planar imaging (EPI) sequence at the same scanner with the following scanning parameters: repetition time (TR) = 2,500 ms, echo time (TE) = 30 ms, flip angle = 90°, number of slices = 40, slice thickness = 3 mm, in-plane resolution = 64×64, number of volumes = 245 or 305. For images that had 305 volumes, we removed the last 60 volumes to make the time point consistent (245 volumes for all images) (44, 45).

### Image Preprocessing

All rs-fMRI data were preprocessed *via* the statistical parametric map 8 (SPM, https://www.fil.ion.ucl.ac.uk/spm/software/spm8) using the general pipeline. Briefly, the pipeline included the following steps: (1) removing the first 5 volumes; (2) correcting slice timing and head motion; (3) registering the functional images to the corresponding T1-weighted images, and normalizing the acquisition to Montreal Neurological Institute (MNI) space with a resampling voxel size of 3×3 × 3 mm^3^ resolution; (4) smoothing the normalized images with a 5-mm full-width at half-maximum (FWHM) Gaussian kernel spatially according to previous literature (46); (5) reducing low-frequency drift and high-frequency noise with bandpass filtering (0.01–0.1 Hz). The global signal regression was not conducted avoiding the removal of the significant neuronal signals (47, 48). Participants with poor image quality or excessive head motion [translation distances > 2 mm or rotation degree > 2° or mean framewise displacement > 0.2 mm (49, 50)] were excluded (51), leaving 30 CBP patients and 29 healthy controls for further analysis. The demographics for patients with CBP and HC are shown in Table 1. The human Brainnetome atlas (52) was used to parcellate the whole brain into 274 regions [246 for the cerebrum and 28 for the cerebellum derived from the Probabilistic Cerebellar Atlas (53)].

**Table 1:** Demographic information of participants.

	**CBP (*N* = 30, mean ±SD)**	**HC (*N* = 29, mean±SD)**	***p*-value**
Age	50.3 ± 8.1	49.2 ± 9.4	0.6519^a^
Gender (M/F)	17/13	17/12	0.6888^b^
BDI score	6.3 ± 5.6	1.3 ± 2.2	<0.05^a^
VAS score	6.8 ± 1.7	-	-
Pain duration	15.9 ± 11.6	-	-

*CBP, chronic back pain; HC, healthy control; BDI, beck depression inventory; VAS, visual analog scale*;

*^a^ denotes two-sided two-sample t-test*;

*^b^ denotes two-sided Pearson chi-square test*.

### Dynamic FC Estimation

**Figure 1:**
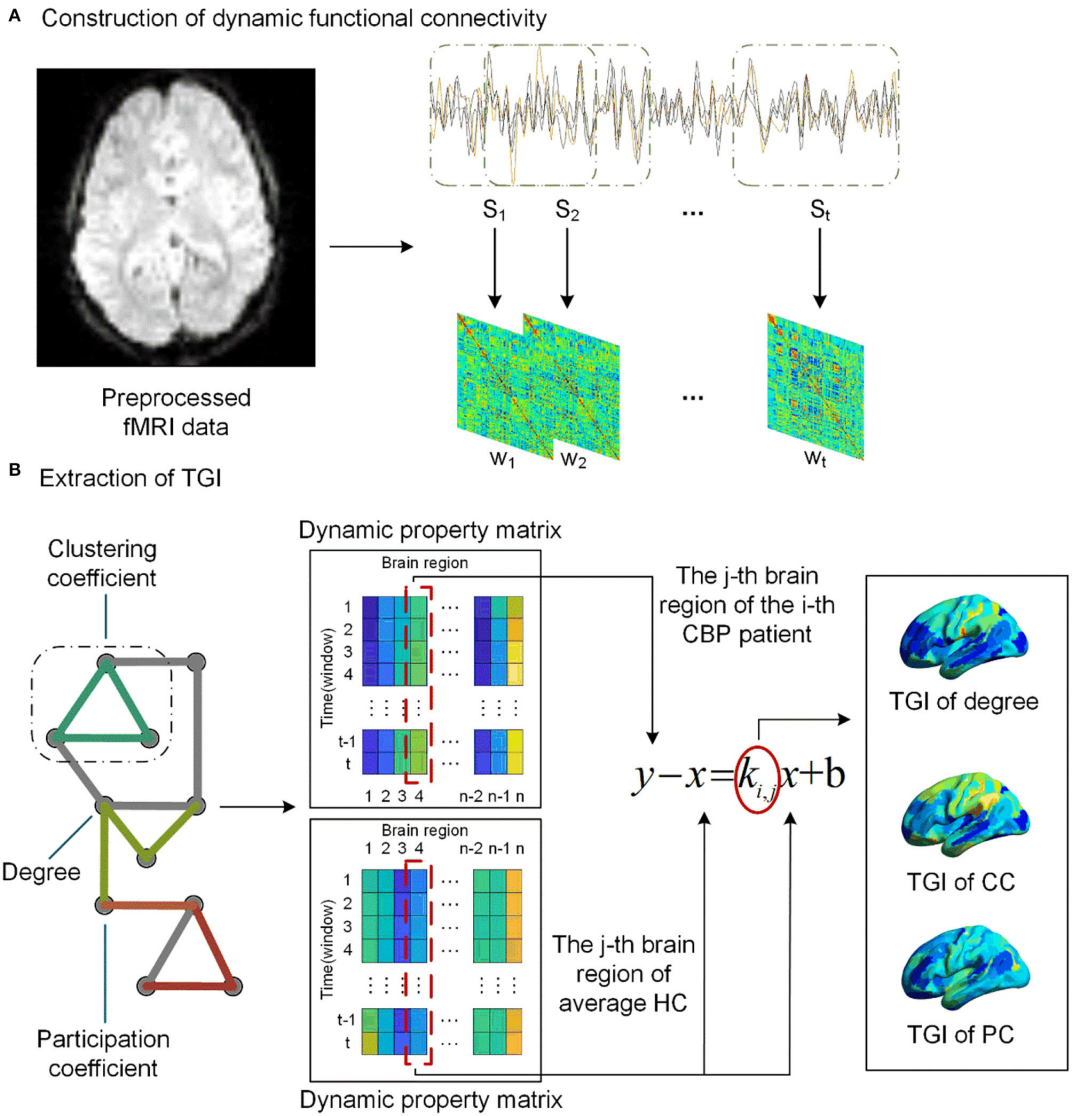
The pipeline of TGI extraction. **(A)** We assess the dynamic functional connectivity (dFC) using the sliding window analysis with 50 TRs length for a window and 1 TR length for sliding step, resulting in t windows. **(B)** We extract dynamic property matrix (degree, clustering coefficient [CC], and participation coefficient [PC]) for the network of each CBP patient and the average network of the HCs. A linear regression model was then used to extract the TGI *K*_*i, j*_ of each network property of the *j*-th brain region for *i*-th CBP subject.

After preprocessing, a data matrix (*n*×*T*) for each participant was obtained, where *T* = 240 denotes the number of time points and *n* = 274 denotes the number of brain regions. We used the DynamicBC toolbox (https://guorongwu.github.io/DynamicBC) (54) to estimate the dynamic functional connectivity (dFC) of each participant. As a key parameter in the sliding window approach, it has been proved that the method would introduce spurious correlations when window lengths < 1/*f*_min_, where *f*_min_ denotes the lowest frequency (i.e., 0.01 Hz) in preprocessing of bandpass filtering (55–57). The dFC matrices were calculated within *t* = 191 consecutive windows produced by sliding window approach with 50 TRs length of the window and 1 TR length of sliding step (58). Finally, *t* functional connectivity matrices *w*∈ℝ^*n*×*n*^ , with negative- and self- connectivity removed (59), were obtained for each participant (Figure 1A).

### Computation of TGI for Patients With CBP

We averaged the functional networks of each time window across the HCs, resulting in a series of average dFC ($W_{h}\in {\mathbb{R}}^{t\times n\times n}$) of HC subjects. The Brain Connectivity Toolbox (BCT, http://www.brain-connectivity-toolbox.net) was used to calculate the network properties of dFC matrices *w* of each subject across time windows, including degree, clustering coefficient (CC), and participation coefficient (PC) (Figure 1B). The PC was calculated based on the modular structure including Yeo's 7 cortical functional networks (i.e., visual, somatomotor, dorsal Attention, ventral attention, limbic, frontoparietal, default and subcortical networks) (60), subcortical network, and cerebellar network. The subcortical network was composed of all subcortical regions, including the amygdala (BN label 211–214), thalamus (BN label 231–246), caudate (BN label 219–220, 227–228), putamen (BN label 225–226, 229–230), globus pallidus (BN label 221–222), nucleus accumbens (BN label 223–224); and the cerebellar network was composed of all cerebellar lobules (excluding the brain stem) in the BN_274 atlas (BN label 247–274). For each network property of a participant, we concatenated this property across time windows to form a dynamic matrix *X*∈ℝ^*t*×*n*^.

As shown in Figure 1B, for the j-th brain region, we subtracted the regional network property calculated from *W*_*h*_ (the averaged dFC networks of the HCs) from the regional network property of each patient with CBP. Then a linear regression (equation 1) model, with the result of the subtraction as the dependent variable and the network property of average HC *W*_*h*_ as the independent variable, was performed to extract the temporal gradient index (TGI) of each brain region of CBP patients.


(1) $$\begin{array}{r} y-x=k_{i,j}x+b \end{array}$$

The slope *k* is the TGI that represents the alteration gradient of this patient relative to the HC group at the temporal scale. For each patient with CBP, we concatenated the TGI of each network property across brain regions. In addition, the combination of all the TGI features was calculated *via* the z-score strategy.

### Regression Analysis

To examine the validity of TGI in assessing the pain intensity of patients with CBP, three commonly used regression models (i.e., support vector regression [SVR], least absolute shrinkage and selection operator [Lasso], and elastic net) were applied to predict pain intensity (VAS scores) using the TGI. Five-fold cross-validation repeated ten times was performed and mean square error (MSE) was used to evaluate the regression performance.

The Linear SVR (61) we used for the prediction can approximate the actual pain intensity y with two hyperparameters E and o ascertain a linear regression function expressed as:


(2) $$\begin{array}{r} f\left(\omega ,b\right)=\omega x+b \end{array}$$

where ω*ϵℝ*^1×*n*^ and b are the parameters of the function. For the prediction of the pain intensity, *xϵℝ*^*n*×1^and the output *f*(*x*_*i*_) denoted the TGI feature of each brain region extracted from patients and the prediction result for the i-th patient, respectively.

The regression problem of Lasso (62) can be described as equation 3, where β*ϵℝ*^*n*×1^ is the parameter of the regression function. The L1-norm regularization was used to make the coefficients sparse so that the irrelevant predictors could be excluded (62).


(3) $$\min_{\beta }\|y-X\beta {\|}^{2}+\lambda \|\beta {\|}_{1}$$

Compared to Lasso, elastic net (63) had one more regularization term (β*ϵℝ*^*n*×1^) and the elastic net will turn into Lasso when setting α =0 (see equation 4). The L2-norm regularization enabled the model to select a subset rather than only one from the highly correlated features to overcome the deficit of using L1-norm regularization only.


(4) $$\min_{\beta }\|y-X\beta {\|}^{2}+\lambda \|\beta {\|}_{1}+\alpha \|\beta {\|}^{2}$$

We used the grid search method to calibrate the λ in Lasso regression according to the previous study (64). For example, the Lasso was constructed by varying the λ in a specified range λ= {0.10,0.15,0.20,...,1} and then the optimal λ was used in the testing dataset. A two-step grid search method was applied to calibrate the parameters for both SVR and elastic net according to previous studies (65, 66). When calibrating parameters of SVR and elastic net models, we first specified a coarse grid search to determine the best region of the calibrated parameters and then conducted a finer grid search to find the optimal parameters.

We then adjusted the bias between predicted pain intensity and real pain intensity according to the bias-adjustment scheme proposed by Beheshti et al. (67). For each subject in the training set, we calculated the Δ by subtracting the real intensity from the predicted intensity of pain and then used a linear regression model of Δ against the real pain intensity to get a linear regression function with the slope μ and the intercept φ. The offset can be calculated as below:


(5) $$\begin{array}{r} offset=\mu \Omega +\varphi \end{array}$$

where Ω denote the real pain intensity. The bias-free back pain intensity was calculated by subtracting the *offset* from individual predicted pain intensity (more information about the relation between Δ and pain intensity could be found in Supplementary Figure S1).

### Statistical Analysis

Between-group differences in age and BDI score were estimated by using a two-sample *t*-test, and the gender difference was estimated *via* the Chi-square test. Pearson correlation analysis was performed to assess the relationship between TGI and BDI score (68) to examine whether the changes in TGI were influenced by affective factors. In addition, a two-sample *t*-test, with age, gender, and BDI score as covariates, was performed to examine the between-group differences in the network properties (i.e., nodal degree, CC and PC) in brain regions that had high prediction power. The false discovery rate (FDR) correction with *q* < 0.05 was used to correct the results for multiple comparisons.

## Result

### Spatial Distribution of the TGI of the Three Network Properties

**Figure 2:**
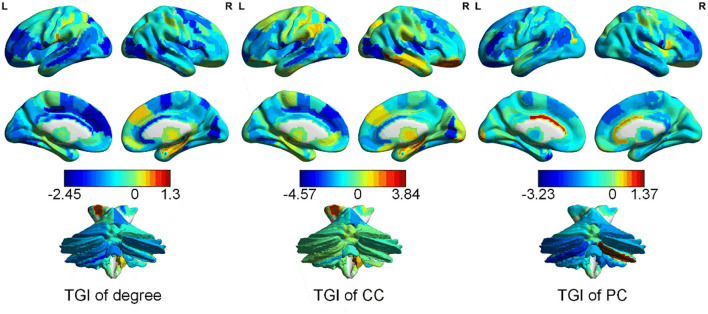
Distribution of the TGI of different network properties [degree, clustering coefficient (CC), and participation coefficient (PC)] in cerebrum and cerebellum.

The average TGI of each network property across patients with CBP is shown in Figure 2. The average TGI of degree is mainly negative values across the brain, except in the left posterior parietal thalamus (PPtha) and right ventrolateral fusiform gyrus [ventrolateral Brodmann area 37 (A37vl)] which have more positive values. Similarly, the average TGI of PC is mostly negative values except for cerebellar right lobule VIIb and left rostroventral ventral anterior cingulate cortex [rostroventral Brodmann area 24 (A24rv)]. The average TGI of CC shows more positive values relative to the other two properties, which locates in A37vl, right rostral temporal thalamus (rTtha), and right medial pre-frontal thalamus (mPFtha), whereas the TGI of medial prefrontal and occipital cortices are highly negative. From the result (more details could be found in Supplementary Figure S2) of Pearson correlation analysis between TGI and BDI scores, we found no significant difference.

### Prediction Performance of Pain Intensity Using the TGI Features

**Figure 3:**
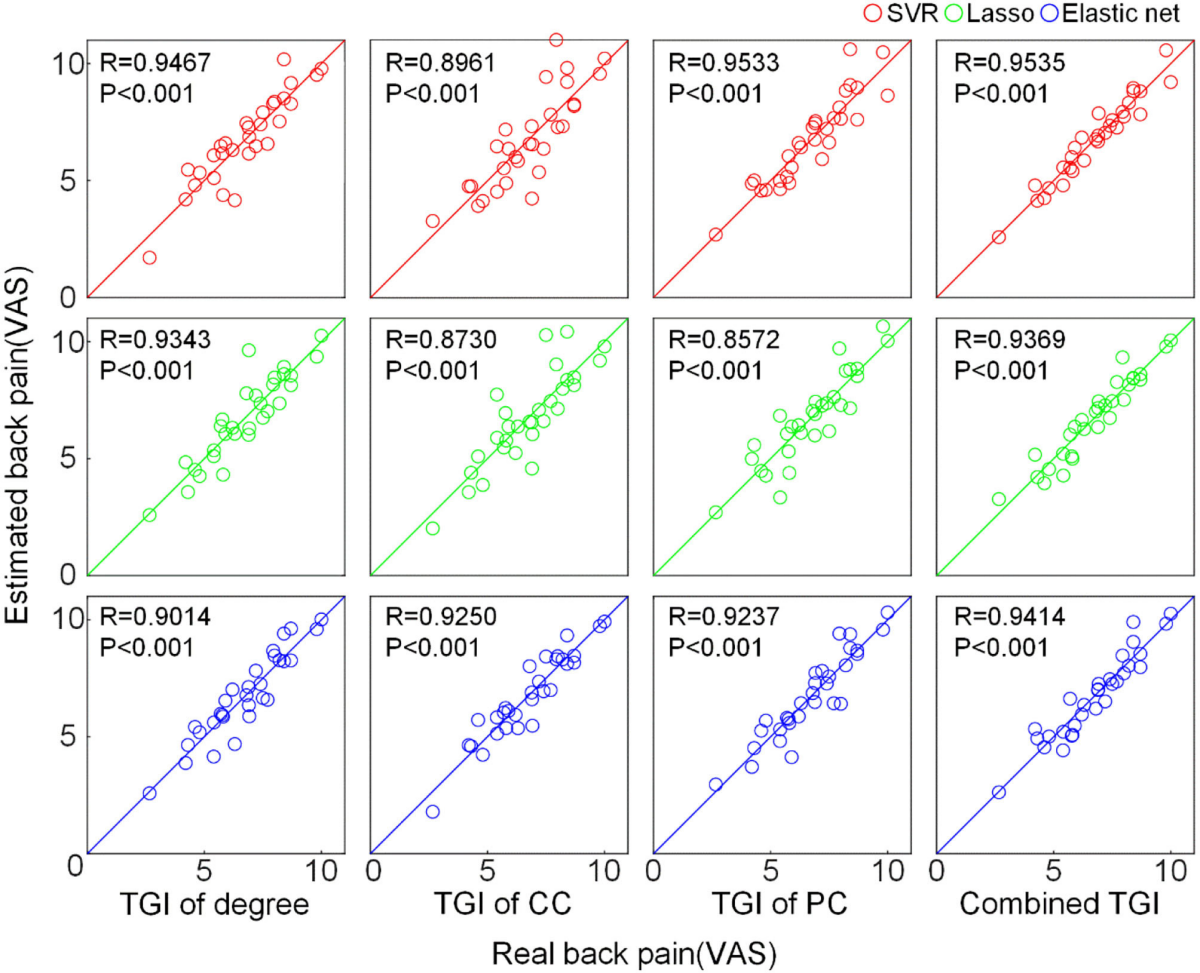
Prediction performance of pain intensity using the TGI of different network properties. The scatter plot shows the correlation between the real VAS score and the predicted VAS score estimated by different features through different regression models. The solid lines indicate the identity line (y = x). CC, clustering coefficient; PC, participation coefficient; VAS, visual analog scale.

**Figure 4:**
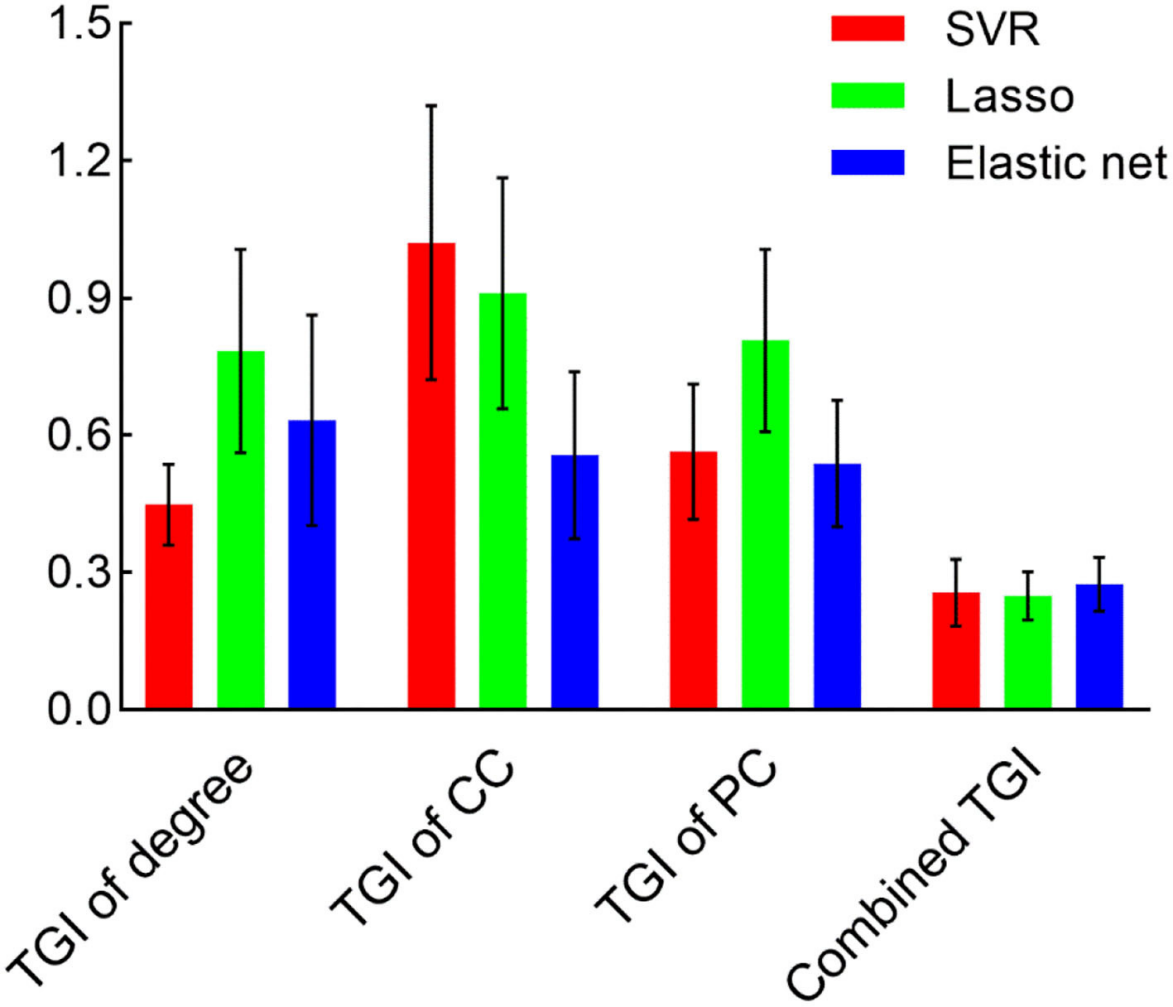
The MSE is derived from different models and features. Bars represent the mean and SD of the MSE during the cross-validation process. CC, clustering coefficient; PC, participation coefficient.

We used the TGI of degree, CC, PC as well as their combination as input features of SVR, Lasso, and elastic net models to predict the VAS score via a cross-validation strategy. The results are visualized in Figure 3. The scatter plots illustrate the correlation between estimation and the real VAS scores. The MSE of each regression task is given in Figure 4. For TGI of degree, SVR achieved the best prediction performance by using the parameters of (O, E) = (1,0.17), with the mean MSE = 0.45 ± 0.09. The TGIs of CC and PC achieved the mean MSEs of 0.56 ± 0.18 and 0.54 ± 0.14, respectively, using the elastic net under the parameters (λ, α) = (0.08,0.28). The combination of all the TGI features significantly improved the regression performance of all the three models, and the Lasso achieved the minimum MSE = 0.25 ± 0.05 under the parameter λ = 0.65.

### Temporal Fluctuation of Network Properties of High Informative Brain Regions

**Figure 5:**
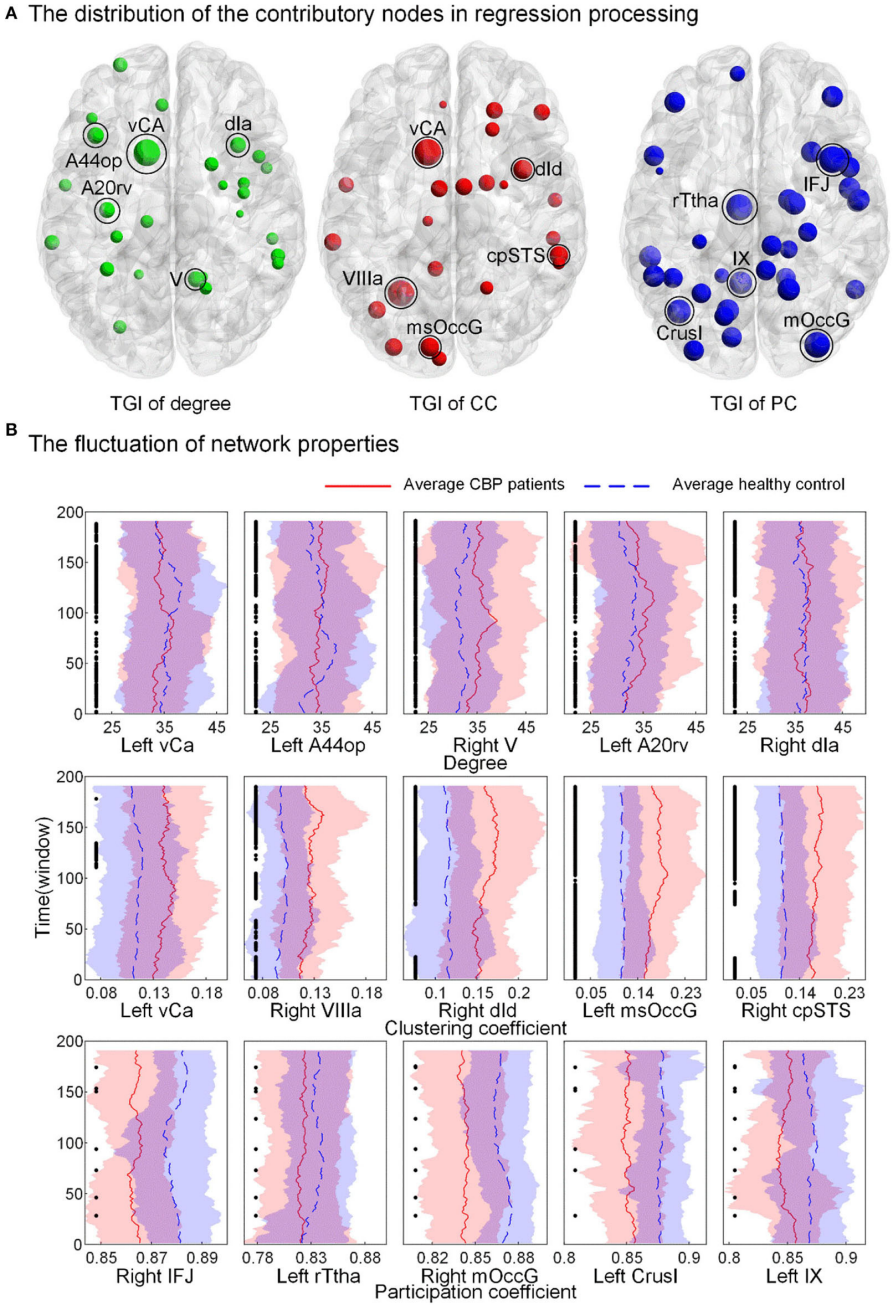
Brain regions have high prediction power and the fluctuation of their nodal topology over time. **(A)** The weight of nodes contributed to the prediction process. Larger nodal size indicates a higher weight. **(B)** The fluctuation of network properties [nodal degree, clustering coefficient (CC), and participation coefficient (PC), not TGI] of the five nodes with the highest weights. The black point indicates a significant between-group difference (*p* < 0.05, FDR corrected). vCa, ventral caudate; A44op, opercular Broca's area (opercular Brodmann area 44); V, cerebellum lobule V; A20rv, rostroventral inferior temporal gyrus (rostroventral Brodmann area 20); dIa, dorsal agranular insula; VIIIa, cerebellum lobule VIIIa; dId, dorsal dysgranular insula; msOccG, medial superior occipital gyrus; cpSTS, caudoposterior superior temporal sulcus; IFJ, inferior frontal junction; rTtha, rostral temporal thalamus; CrusI, cerebellum lobule Crus I; IX, cerebellum lobule IX.

The distribution of brain regions with the TGI that highly contributed to the regression process is shown in Figure 5A (the fluctuations of the other brain regions can be found in Supplementary Figures S3–S5 in Supplementary Materials). For the TGI of degree, brian regions contributing to prediction were mainly placed in the temporal lobe, subcortical nuclei, and insular cortex, including superior temporal gyrus (STG), insular gyrus (INS), and basal ganglia (BG). For the TGI of CC, the high informative brain regions were located in the frontal and temporal lobes, including the superior frontal gyrus (SFG), superior temporal gyrus (STG), and inferior temporal gyrus (ITG). For the TGI of PC, high-weight brain regions were located across frontal, temporal, and parietal cortices, including the middle frontal gyrus (MFG), posterior superior temporal sulcus (pSTS), inferior frontal gyrus (IFG), superior temporal gyrus (STG) and superior parietal lobule (SPL). Furthermore, the cerebellum, caudoposterior superior temporal sulcus (cpSTS), and rostral somatosensory association cortex [rostral Brodmann area 7 (A7r)] highly contributed to regression tasks using each type of TGI. We also compared the fluctuations of degree, CC, and PC of five brain regions, including ventral caudate (vCa), opercular Broca's area [opercular Brodmann area 44 (A44op)], cerebellum lobule V (V), rostroventral inferior temporal gyrus [rostroventral Brodmann area 20 (A20rv)], dorsal agranular insula (dIa), cerebellum lobule VIIIa (VIIIa), dorsal dysgranular insula (dId), medial superior occipital gyrus (msOccG), caudoposterior superior temporal sulcus (cpSTS), inferior frontal junction (IFJ), rostral temporal thalamus (rTtha), cerebellum lobule Crus I (CrusI) and cerebellum lobule IX (IX), with the highest weights in pain prediction between the two groups (Figure 5B). Compared to the HC cohort, the CC showed higher values over time in patients with CBP in all the five brain regions, whereas, the PC in these regions showed the opposite alteration trend in the CBP cohort. In addition, the fluctuations of the nodal degree of CBP patients showed a relatively larger overlap with the HC when compared to the other two network properties.

## Discussion

The present study aimed to (1) investigate whether CBP is associated with altered dynamics of topological organization of functional brain networks, and (2) develop a novel kind of feature (TGI) that could better characterize pain sensation from dynamic functional networks. We found CBP significantly altered the dynamics of functional network properties, and the gradient of these dynamics of CBP patients relative to the HCs accurately predicted pain intensity. These results suggested that CBP is accompanied by abnormal alterations of functional topology at the temporal scale, which may serve as an effective biomarker for estimating pain perception in the brain.

Studies have shown that the degree gradient of patients with CBP relative to the HCs can characterize a unique neurological state of chronic pain (46, 69), such as a global randomization state of functional connectivity (46). Regarding the dynamic nature of the functional brain connectome, we speculated that the TGI extracted from the dFC networks might better depict the variations in neurological states of CBP over time than using static functional networks. The high prediction performance of pain intensity suggested the effectiveness of the TGI feature, and no significant difference between TGI and BDI scores suggested the stabilization of the TGI feature that TGI would not be influenced by abnormal emotion (i.e., depression). Furthermore, the combination of three types of TGI significantly improved the prediction performance, suggesting the complex neural mechanism of CBP requires information from diversified domains to assess pain sensation. Nodal degree, CC and PC were indicated to represent network hubness, segregation, and integration of the brain network, respectively (59, 70, 71). Studies have reported the differences in nodal degree and PC of functional brain networks between patients with CBP and the HCs (72, 73). The CC of the insular cortex was found to be correlated with individual pain thresholds (74). Therefore, the combination of the TGI of these three network properties can comprehensively depict the alternation of brain networks of patients with CBP, which undoubtedly performed better than the TGI of a single network property in predicting the pain intensity.

Interestingly, we also found that the dynamics of CC and PC of brain regions that had TGI with high prediction power (i.e., STG, ITG, pSTS, SPL, IFG, INS, and parts of the cerebellum) showed distinct fluctuation patterns between patients with CBP and the HCs. Although previous studies have shown altered CC and PC of the static functional network in the CBP cohort (70, 75), our study moved a further step to show abnormalities in the temporal fluctuation of these two properties in patients with CBP. Since CC and PC represented the segregation and integration of the network, respectively. The altered fluctuation of them may indicate the topological reorganization of functional brain networks in patients with CBP that the network tended to be more locally connected with disruptions in inter-modular connectivity. This is in line with previous studies indicating lower efficiency of information transfer in the brain networks of patients with CBP than the HCs (42, 76). The brain regions with abnormal fluctuation of CC and PC were indicated to be extensively involved in pain processing (77–80). For example, IFG and ITG are involved in pain-related memories (81, 82), and INS plays a critical role in pain modulation (83). Furthermore, the cpSTS and the A7r of SPL showed a high contribution to pain prediction in all regression tasks using the TGI derived from different network properties (i.e., degree, CC and PC), suggesting the abnormal changes in these two brain regions were not only in local connectivity with other regions but also in the flow of information throughout the brain. These findings were supported by the previous studies indicating increased vigilance of the pain within these two brain regions (84–86). All these results supported our argument that TGI of network properties might better characterize the neurological condition of the individual with CBP in a dynamic manner.

There were several limitations in the present study. First, the sample size was limited in this study. Here, we performed a 5-fold cross-validation strategy and employed three commonly used regression models (i.e., SVR, Lasso and elastic net) that showed high generalizability across studies (87–89) to reduce the risk of overfitting, and achieved robust performance. Replications on a larger and independent dataset are still necessary to further verify the effectiveness of TGI of network topology in assessing pain intensity. Second, the dynamics of functional networks largely rely on the chosen parameters of the sliding window approach that determine the scale of the time sequence (90). In the present study, we chose the parameters according to the previous literature (55–57). Nevertheless, whether the parameters could influence the prediction power of TGI on the pain intensity need to be further explored.

## Conclusion

We proposed a novel feature called TGI that was derived from the dFC network to represent the temporal deviation of network topology in patients with CBP relative to HCs. The TGI of network properties achieved outstanding performance in predicting the pain intensity of patients with CBP in three commonly used regression models, with a minimum MSE of 0.25 ± 0.05. Our findings suggested that the TGI can serve as a valuable biomarker for pain intensity evaluation and has potential application in CBP management/therapy.

## Data Availability Statement

The datasets presented in this study can be found in online repositories. The names of the repository/repositories and accession number(s) can be found at: https://www.openpain.org/.

## Ethics Statement

The studies involving human participants were reviewed and approved by Northwestern University's Institutional Review Board Committee. The patients/participants provided their written informed consent to participate in this study. Written informed consent was obtained from the individual(s) for the publication of any potentially identifiable images or data included in this article.

## Author Contributions

ZL drafted the manuscript. LZ conducted data analysis. ZZ polished the manuscript. All authors provided feedback and revised the manuscript.

## Funding

This work is supported by the Science and Technology Innovation 2030-Major Project of China (2021ZD0202002) and the China Postdoctoral Science Foundation (2020M671726).

## Conflict of Interest

The authors declare that the research was conducted in the absence of any commercial or financial relationships that could be construed as a potential conflict of interest.

## Publisher's Note

All claims expressed in this article are solely those of the authors and do not necessarily represent those of their affiliated organizations, or those of the publisher, the editors and the reviewers. Any product that may be evaluated in this article, or claim that may be made by its manufacturer, is not guaranteed or endorsed by the publisher.
