# Supplementary material for: Temporal Grading Index of Functional Network Topology Predicts Pain Perception of Patients With Chronic Back Pain
Source: Front Neurol. 2022 Jun 10;13:899254. doi: 10.3389/fneur.2022.899254 (PMC9226296; doi:10.3389/fneur.2022.899254)
Supplement: Supplementary file 1 [file Table_1.DOCX]

Supplementary Figures and Legends

Figure

 S1. Example of the pain-related bias in back pain delta in our data, where the delta is the estimated back pain minus the real back pain (real VAS score). The dashed red line shows the reference line (y=0), while the solid blue line states the regression line. In this scheme, we first calculated the $\Delta$ by subtracting the real intensity from the predicted pain intensity for each subject in the training set. Then a linear regression model of $\Delta$ against the real pain intensity was performed to get a linear regression function with the slope $\mu$ and the intercept $\varphi$. The offset can be calculated with , where $\Omega$ denote the real pain intensity. The bias-free back pain intensity was calculated by subtracting the offset from individual predicted pain intensity. As can be seen, there is a significant dependence of the back pain delta on real back pain (real VAS score) ($r=-0.85, p<0.001;y=-0.94x+6.88$). And then the bias can be adjusted by the bias-adjustment scheme.



Figure S2. The distribution of $1-p$ across the whole brain, where the values of $p$ denotes the results of p-values (FDR corrected) of Pearson correlation analysis between TGI and BDI score. The result of Pearson correlation analysis shows no significant difference.



Figure S3. The validation of the predictive power of the TGI of the other network properties (e.g., betweenness centrality [BC] and global efficiency [GE]). (A) The performance of the TGI of the BC and GE in back pain prediction. The scatter plot shows the correlation between the real VAS score and the predicted VAS score estimated by different features through different regression models. The solid lines indicate the identity line (y=x). (B) The MSE derived from different models and features. Bars represent the mean and SD of the MSE during cross-validation process. The results shown in Figure S3 indicate the predictive power of the TGI of BC and GE.





Figure S4. The fluctuations of nodal degree of the other brain regions. The black point indicates a significant between-group difference ($p<0.05$, FDR corrected). A22c, caudal superior temporal gyrus (caudal Brodmann area 22); A22r, rostral superior temporal gyrus (rostral Brodmann area 22); dmPOS, dorsomedial parietooccipital sulcus; A5l, lateral somatosensory association cortex (lateral Brodmann area 5); cHipp, caudal hippocampus; CrusII, cerebellum lobule Crus II; A10l, lateral frontopolar area (lateral Brodmann area 10); VIIIb, cerebellum lobule VIIIb; cTtha, caudal temporal thalamus; A32sg, subgenual dorsal anterior cingulate cortex (subgenual Brodmann area 32); A7r, rostral somatosensory association cortex (rostral Brodmann area 7); vmPu, ventromedial putamen; lAmyg, lateral amygdala; G, hypergranular insula; dIg, dorsal granular insula; vId/vIg, ventral dysgranular and granular insula; A38l, lateral temporopolar area (lateral Brodmann area 38); A21r, rostral middle temporal gyrus (rostral Brodmann area 21); A20cv, caudoventral inferior temporal gyrus (caudoventral Brodmann area 20); cpSTS, caudoposterior superior temporal sulcus; A40c, caudal supramarginal gyrus part of Wernicke's area (caudal Brodmann area 40).





Figure S5. The fluctuations of clustering coefficient of the other brain regions. The black point indicates a significant between-group difference ($p<0.05$, FDR corrected). A22c, caudal superior temporal gyrus (caudal Brodmann area 22); A22r, rostral superior temporal gyrus (rostral Brodmann area 22); CrusI, cerebellum lobule Crus I; iOccG, inferior occipital gyrus; VI, cerebellum lobule VI; cTtha, caudal temporal thalamus; A23v, ventral posterior cingulate cortex (ventral Brodmann area 23); A6m, medial pre-motor and supplementary motor cortex (medial Brodmann area 6); cCunG, caudal cuneus gyrus; A32sg, subgenual dorsal anterior cingulate cortex (subgenual Brodmann area 32); A7r, rostral somatosensory association cortex (rostral Brodmann area 7); mAmyg, medial amygdala; A8dl, dorsolateral frontal eye field (dorsolateral Brodmann area 8); A11l, lateral orbitofrontal area (lateral Brodmann area 11); lAmyg, lateral amygdala; IFS, inferior frontal sulcus; A20cv, caudoventral inferior temporal gyrus (caudoventral Brodmann area 20); A20il, intermediate lateral inferior temporal gyrus (caudoventral Brodmann area 20); A40c, caudal supramarginal gyrus part of Wernicke's area (caudal Brodmann area 40).





Figure S6. The fluctuations of participation coefficient of the other brain regions. The black point indicates a significant between-group difference ($p<0.05$, FDR corrected). The full name of the brain regions shown in the figure can be found in Table S1. mOccG, middle occipital gyrus; rpSTS, rostroposterior superior temporal sulcus; cpSTS, caudoposterior superior temporal sulcus; A44v, ventral pars opercularis Broca's area (ventral Brodmann area 44); A6cvl, caudal ventrolateral pre-motor and supplementary motor cortex (caudal ventrolateral  Brodmann area 6); A37lv, lateroventral fusiform gyrus (ventral Brodmann area 37); A9/46v, ventral dorsolateral prefrontal cortex (ventral Brodmann area 9/46); TL, area TL (lateral PPHC, posterior parahippocampal gyrus); A7ip, intraparietal somatosensory association cortex (intraparietal Brodmann area 7); VIIIb, cerebellum lobule VIIIb; A7c, caudal somatosensory association cortex (caudal Brodmann area 7); vmPOS,ventromedial parietooccipital sulcus; cLinG, caudal lingual gyrus; A10m, medial frontopolar area (medial Brodmann area 10); A5m, medial somatosensory association cortex (medial Brodmann area 5); A1/2/3ll, lower limb region; A7r, rostral somatosensory association cortex (rostral Brodmann area 7); TH, area TH (medial PPHC); A28/34, entorhinal cortex; rHipp, rostral hippocampus; cHipp, caudal hippocampus; A38m, medial temporopolar area (medial Brodmann area 38); iOccG, inferior occipital gyrus; A38l, lateral temporopolar area (lateral Brodmann area 38); A37vl, ventrolateral fusiform gyrus (ventral Brodmann area 37); A20il, intermediate lateral inferior temporal gyrus (caudoventral Brodmann area 20); A1/2/3tonIa, tongue and larynx region.
